# Association Between Function-focused Training and Physical Function Among Community-dwelling Oldest-old Individuals: A Retrospective Study

**Published:** 2026-08-05

**Authors:** MUNETOSHI KAWAKAMI, AIKO ISHIKAWA, MORITOMO MAEDA, MAMI TANI, REINA ISAYAMA, FUTOSHI WADA, AKIRA TANUMA, KAORU HONAGA, TOSHIYUKI FUJIWARA

**Affiliations:** 1 Department of Rehabilitation Medicine, Juntendo University Graduate School of Medicine, Tokyo, Japan; 1 Department of Rehabilitation Medicine, Juntendo University Graduate School of Medicine, Tokyo, Japan; 2 Department of Physical Therapy, Juntendo University Faculty of Health Science, Tokyo, Japan; 2 Department of Physical Therapy, Juntendo University Faculty of Health Science, Tokyo, Japan

**Keywords:** community dwelling, aged, physical performance, preventive health services, rehabilitation

## Abstract

**Objective:**

To examine the 12-month relationship between physical function-focused training and lower-limb function among community-dwelling elderly.

**Design:**

In this single-center retrospective cohort study, 93 adults aged ≥ 65 years (median age 85.0 years; 74.2% women) who attended a function-focused day service at least once per week and completed the Short Physical Performance Battery (SPPB) at baseline and 12 months were included.

**Methods:**

Each 105-minute session consisted of 80 minutes of group training for flexibility, strength, and balance, and 25 minutes of individualized machine-based aerobic, resistance, and flexibility training. The primary outcome was the 12-month change in SPPB total score; secondary outcomes included SPPB subscores and grip strength. Participants were categorized according to change in SPPB as Improved (≥ +1 point), Maintained (0), or Deteriorated (≤ -1 point).

**Results:**

Median SPPB total score remained stable (10.0 [7.0-12.0] to 10.0 [7.5-12.0]; Hodges-Lehmann change, 0.0 [0.0-0.5]; p =  0.76); 34 participants (36.6%) improved, 32 (34.4%) maintained, and 27 (29.0%) deteriorated. The Improved group showed increases in balance and chair-stand scores, while all subscores declined in the Deteriorated group. Grip strength decreased significantly in the total group but remained stable within the Improved group.

**Conclusions:**

These findings suggest that low-frequency, low-intensity function-focused training performed at least once per week may help maintain lower-limb function, even among community-dwelling oldest-old individuals.

## Introduction

Maintaining physical function is a fundamental component of healthy aging and is essential for elderly individuals to continue living independently in their familiar communities. Among community- dwelling elderly, lower-limb functional decline is strongly associated with the loss of independence, an increased risk of falls, institutionalization, and higher mortality^1-5)^. Maintenance of physical function contributes not only to individual quality of life but also to broader socioeconomic benefits such as reducing medical and long-term care costs^6-8)^.

Based on numerous international studies of community-dwelling elderly, it is well established that maintaining regular training habits and an adequate level of physical activity is indispensable for preserving and improving physical function^9-12)^.

However, previous studies have several limitations. First, there is a bias in the age range of participants, as most existing research has focused on relatively healthy elderly individuals with mean ages in their 70s^4, 9, 10, 13)^. Very few studies have targeted very old community-dwelling individuals aged 80 years or older, despite their higher risk of mobility loss and functional decline^14, 15)^.

Second, the follow-up periods in prior research have often been short, with many intervention studies examining the effects over only 3-6 months^16, 17)^. Accordingly, there is insufficient evidence regarding the long-term effects of training interventions lasting 12 months or longer.

Third, the frequency of training interventions has been limited mainly to high-frequency programs conducted two to three times per week^18-20)^. Consequently, the long-term effects of lower-frequency interventions of approximately once weekly, which is a more realistic and sustainable frequency for very old elderly, remain unclear.

In addition, there are methodological issues in evaluating training exposure. Many cohort studies have relied on self-reported physical activity levels (e.g., low, moderate, or high; exerciser vs. non-exerciser), which are susceptible to recall and social desirability biases^21, 22)^. Such measures may not accurately reflect the actual frequency of training and may obscure dose-response relationships. Moreover, most previous studies have analyzed mean values or event rates (e.g., falls, mortality), with few reporting the proportions of improvement, maintenance, and deterioration^23)^.

Therefore, the objective of this retrospective study was to address the limitations of previous studies by examining the outcomes of a 12-month, function-focused training program. The program was delivered in 105-minute sessions at least once weekly within a day-service facility for a cohort primarily composed of community-dwelling oldest- old individuals.

## Materials and Methods

### Study design and ethical approval

This was a single-facility retrospective cohort study conducted in accordance with the STROBE guidelines. Data were collected between May 1, 2023, and July 31, 2024, at a function-focused day service facility in Tokyo, Japan. All data were routinely collected as part of standard clinical operations, anonymized before analysis, and contained no missing values. The study protocol was approved by the Ethics Review Committee of Juntendo University (Approval No. E24-0274). Information about the study was publicly disclosed on the facility and university websites, and participants were allowed to opt out if they did not wish to take part.

### Participants

**Figure 1:**
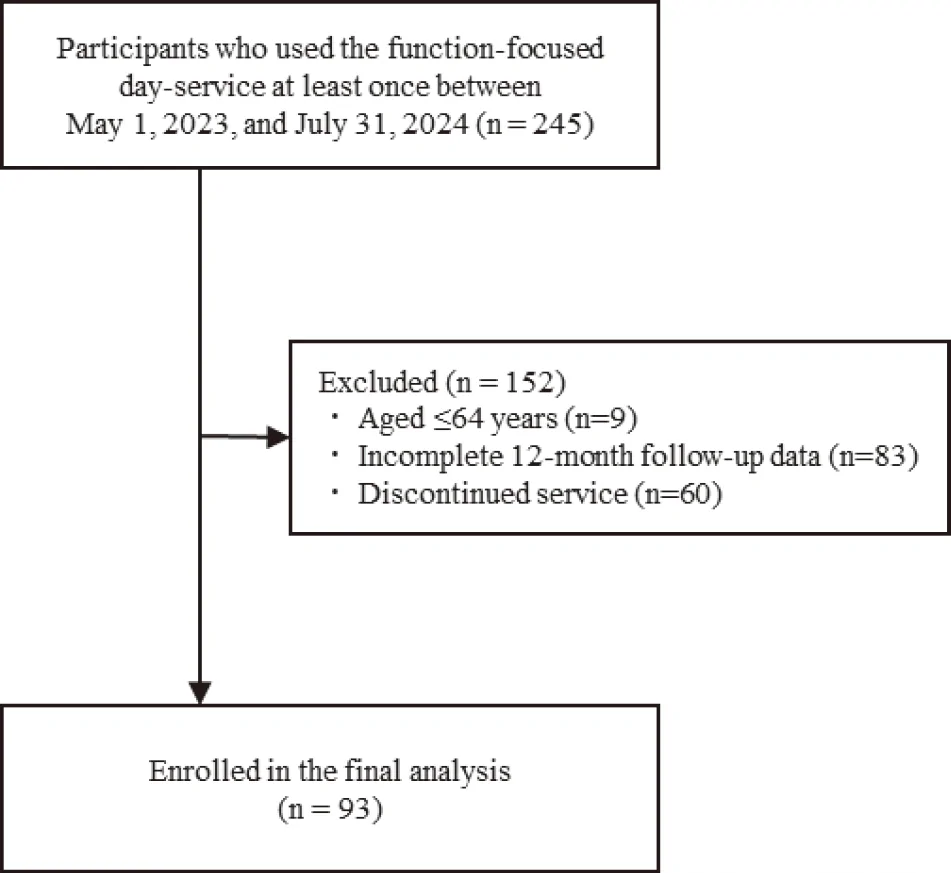
Study flow diagram

Eligible participants were community-dwelling elderly aged 65 years or older who required some level of support with daily activities and were enrolled in a physical function-focused day-service program following a formal care planning process, in which certified care professionals determined that maintaining or improving physical function was necessary to sustain daily life. Participants had continuously attended the program at least once per week, were able to understand the assessment procedures, and completed physical function assessments at both baseline and after 12 months of attendance. A total of 93 participants were included in the final analysis (69 women, 74.2%; median age, 85.0 [interquartile range, 80.5-89.0] years) (Figure 1). All participants lived in the community with varying levels of support. The level of required support varied, ranging from mild assistance with selected instrumental activities of daily living, such as shopping or meal preparation, to more extensive support across daily activities; however, the study population primarily consisted of individuals who required only mild assistance with some basic activities of daily living. Table 1 summarizes the participant characteristics.

**Table 1:** Characteristics of the participants

Characteristics (n = 93)	Values
Age (years)	85.0 [80.5-89.0]
Women, n(%)	69 (74.2)
Day-service attendance (days/week)	1.0 [1.0-2.0]
Service use duration (months)	14.0 [8.0-20.0]

a) Values are presented as median [interquartile range] for continuous variables, and number (%) for categorical variables. b) Service use duration is the period (months) during which the participants used a function-focused day-service before the baseline.

### Physical function-focused training program

The physical function-focused training program was conducted under the supervision of physical therapists and other trained professionals and lasted 105 minutes per session. Each session consisted of approximately 80 minutes of group-based training and 25 minutes of individualized machine-based training. Group-based training included a structured warm-up (stretching and mild upper- and lower-limb training), orofacial movements, flexibility training, and suspension-based training. Suspension- based training accounted for the largest proportion of the group training time and was performed in chair-seated and standing positions, primarily targeting lower-limb muscle strength and postural control through static and dynamic balance tasks. Individualized training comprised short bouts of aerobic, resistance, and flexibility training, including treadmill walking, recumbent cycling, leg extension training using a machine, whole-body vibration exercises, and machine stretching focusing on the hip and trunk muscles. Although a gait training device such as a treadmill was used for short periods, the program primarily emphasized balance and lower-limb strength training. For safety considerations, including fall prevention, task-specific or complex gait training—such as obstacle negotiation, fast-paced walking, or frequent direction changes—was not incorporated into the program. Participants attended the program regularly at least once per week, and attendance was monitored monthly through facility usage records.

### Assessments and outcomes

Physical function was assessed at baseline and after 12 months using the Short Physical Performance Battery (SPPB) and grip strength. The SPPB evaluates lower-limb function through three components (static balance, 4-meter gait speed, and five-time chair stand) and provides a total score ranging from 0 to 12, with higher scores indicating better performance^2)^. The SPPB has been validated as a reliable measure of lower-limb function and is associated with disability incidence and mortality risk^3-5)^. Grip strength was measured using a handheld dynamometer (Grip D; Toei Light Co., Ltd., Tokyo, Japan) while standing with the arm extended alongside the trunk. Each hand was tested twice, and the highest value (kg) was used for analysis.

The primary outcome was the change in SPPB total score over 12 months, and the secondary outcomes were changes in SPPB subscores and grip strength. Participants were further classified into three groups according to the 12-month change in SPPB total score: the Improved group (an increase of one point or more), the Maintained group (no change in total score), and the Deteriorated group (a decrease of one point or more). Baseline characteristics and within-group changes over 12 months were then analyzed for each group.

### Statistical analysis

Continuous variables were tested for normality and summarized as medians [interquartile ranges], and categorical variables were expressed as counts (percentages). Changes from baseline to 12 months were analyzed using the Wilcoxon signed-rank test, and effect sizes were expressed as Hodges-Lehmann estimates (ΔHL) with 95% confidence intervals. Between-group comparisons were performed using the Kruskal-Wallis test for continuous or ordinal variables, followed by post hoc pairwise comparisons with Bonferroni correction when appropriate. Categorical variables were compared using the χ^2^ test. A two-tailed p-value < 0.05 was considered statistically significant. All analyses were performed using IBM SPSS Statistics (version 29.0; IBM Corp., Armonk, NY, USA) and EZR (Jichi Medical University, Tochigi, Japan)^24)^.

## Results

The median age of the 93 participants was 85.0 [80.5-89.0] years, and 69 (74.2%) were women (Table 1). Table 2 summarizes changes in physical function over 12 months for the entire cohort. The median SPPB total score remained stable, changing from 10.0 [7.0-12.0] at baseline to 10.0 [7.5-12.0] at 12 months (ΔHL 0.0 [0.0 to 0.5]; p = 0.76). All SPPB subscores were maintained, although gait speed showed a marginally significant trend (p = 0.05), suggesting individual variability in change. Grip strength significantly decreased from 18.8 to 17.7 kg (ΔHL −0.9 [−1.4 to −0.4]; p < 0.05). The frequency of day-service attendance did not change substantially during the study period (1.0 [1.0-2.0]; p = 0.32).

Based on the 12-month change in SPPB total score, participants were classified into three groups: Improved (n = 34, 36.6%), Maintained (n = 32, 34.4%), and Deteriorated(n = 27, 29.0%)(Table 3). No significant differences in baseline characteristics were observed among the three groups.

**Table 2:** Comparison of participants' physical function assessments between baseline and 12 months

Assessments (n = 93)	Baseline	12 months	ΔHL (95% CI)	p-value
SPPB				
Total (0-12)	10.0 [7.0-12.0]	10.0 [7.5-12.0]	0.0 (0.0 to 0.5)	0.76
Balance (0-4)	4.0 [3.0-4.0]	4.0 [2.0-4.0]	0.0 (0.0 to 0.0)	0.90
Gait speed (0-4)	4.0 [3.0-4.0]	4.0 [3.0-4.0]	0.0 (0.0 to 0.0)	0.05
Chair stand (0-4)	3.0 [2.0-4.0]	3.0 [2.0-4.0]	0.0 (0.0 to 0.0)	0.88
Grip strength (kg)	18.8 [15.2-24.8]	17.7 [14.6-23.0]	−0.9 (−1.4 to −0.4)	< 0.05

a) Values are presented as median [interquartile range] for continuous variables. b) p-values are derived from the Wilcoxon signed-rank test comparing baseline and 12-month values. c) ΔHL (95% CI), Hodges-Lehmann median change (12 months − baseline) with 95% confidence interval; SPPB, Short Physical Performance Battery.

**Table 3:** Between-group comparison of characteristics based on SPPB total score 12-month change

Characteristics	Improved (n = 34; 36.6%)	Maintained (n = 32; 34.4%)	Deteriorated (n = 27; 29.0%)	p-value
Age (years)	84.5 [81.0-90.3]	83 [78.0-88.0]	86 [82.0-89.0]	0.21
Women, n (%)	27 (79.4)	22 (68.8)	20 (74.1)	0.61
Day service attendance (days/week)	1.0 [1.0-1.25]	1.0 [1.0-2.0]	1.0 [1.0-1.0]	0.71
Service use duration (months)	17.0 [10.25-20.0]	14.0 [8.0-17.0]	16.0 [11.0-23.0]	0.11

a) Data are expressed as median [interquartile range] for continuous variables and as number (%) for categorical variables. b) Between-group p-values at baseline: Kruskal-Wallis (continuous), χ^2^ (categorical). c) Service use duration is the period (months) during which the participants used a function-focused day-service before the baseline.

**Figure 2:**
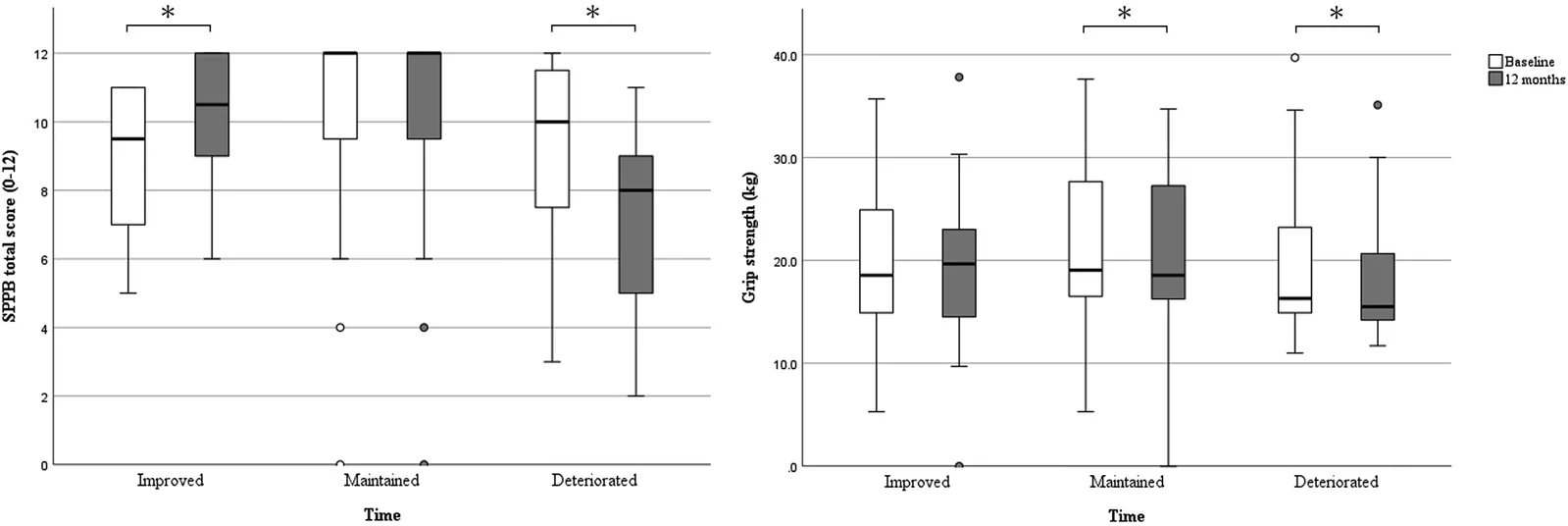
Changes in SPPB total score and grip strength in the improved, maintained, and deteriorated groups Group definitions: Improved = ΔSPPB ≥ +1; Maintained = ΔSPPB = 0; Deteriorated = ΔSPPB ≤ −1 (ΔSPPB = 12-month score - baseline score). Improved group, n = 34 (36.6%); Maintained group, n = 32 (34.4%); Deteriorated group, n = 27 (29.0%). SPPB, Short Physical Performance Battery *p < 0.05 (Wilcoxon signed-rank test)

Table 4 summarizes the 12-month changes in physical function within each group, and Figure 2 illustrates the changes in SPPB total score and grip strength. The median SPPB total score significantly increased in the Improved group (from 9.5 [6.8-11.0] to 10.5 [8.8-12.0]; p < 0.05), remained stable in the Maintained group (from 12.0 [9.3-12.0] to 12.0 [9.3-12.0]), and significantly decreased in the Deteriorated group (from 10.0 [7.0-12.0] to 8.0 [5.0-9.0]; p < 0.05). Regarding SPPB sub-scores, balance and chair-stand performance increased significantly in the Improved group, whereas all sub-scores significantly declined in the Deteriorated group. Grip strength remained stable in the Improved group but significantly decreased in both the Maintained and Deteriorated groups.

**Table 4:** Within-group changes in assessments over 12 months by the SPPB-change group

Assessments	Group	Baseline	12 Months	ΔHL (95% CI)
SPPB Total (0-12)	Improved	9.5 [6.8-11.0]	10.5 [8.8-12.0]	+1.5 (1.0 to 1.5)*
	Maintained	12.0 [9.3-12.0]	12.0 [9.3-12.0]	0.0 (0.0 to 0.0)
	Deteriorated	10.0 [7.0-12.0]	8.0 [5.0-9.0]	-2.0 (-2.5 to -1.5)*
Balance (0-4)	Improved	3.0 [2.0-4.0]	4.0 [3.0-4.0]	+0.5 (0.5 to 1.0)*
	Maintained	4.0 [2.5-4.0]	4.0 [3.3-4.0]	0.0 (0.0 to 0.0)
	Deteriorated	4.0 [3.0-4.0]	2.0 [2.0-4.0]	-1.0 (-1.0 to -0.5)*
Gait speed (0-4)	Improved	4.0 [2.0-4.0]	4.0 [3.0-4.0]	0.0 (0.0 to 0.5)
	Maintained	4.0 [4.0-4.0]	4.0 [4.0-4.0]	0.0 (0.0 to 0.0)
	Deteriorated	4.0 [3.0-4.0]	3.0 [2.0-4.0]	-0.5 (-0.5 to 0.0)*
Chair stand (0-4)	Improved	3.0 [1.8-3.0]	4.0 [2.0-4.0]	+0.5 (0.5 to 1.0)*
	Maintained	4.0 [3.0-4.0]	4.0 [3.0-4.0]	0.0 (0.0 to 0.0)
	Deteriorated	3.0 [1.0-4.0]	1.0 [1.0-3.0]	-0.5 (-1.0 to -0.5)*
Grip strength (kg)	Improved	18.6 [14.8-25.0]	19.7 [14.4-23.7]	-0.4 (-1.2 to 0.3)
	Maintained	19.1 [16.2-27.9]	18.6 [16.2-28.3]	-1.0 (-1.9 to -0.1)*
	Deteriorated	16.3 [14.7-23.8]	15.5 [14.1-21.7]	-1.2 (-2.2 to -0.3)*

a) Improved group, n = 34; Maintained group, n = 32; Deteriorated group, n = 27. b) Data are expressed as median [interquartile range] for continuous variables. c) Within-group changes from baseline to 12 months were analyzed using the Wilcoxon signed-rank test. d) ΔHL (95%CI), Hodges-Lehmann median change (12 months - baseline) with 95% confidence interval; SPPB, Short Physical Performance Battery. e) *p < 0.05 versus baseline of each group.

At baseline, group comparisons revealed significant differences in the total SPPB score and chair-stand score, with the Maintained group showing higher scores than both the Improved and Deteriorated groups (p < 0.05). In addition, balance scores were higher in the Maintained group than in the Improved group (p < 0.05). Overall, the Maintained group showed superior total and subscore performance compared with the other two groups, whereas no significant differences were observed between the Improved and Deteriorated groups. There were no significant differences in grip strength among the three groups (p = 0.37) or by sex (men, p = 0.38; women, p = 0.61). However, in both men and women, median grip strength was highest in the Maintained group, followed by the Improved group, and lowest in the Deteriorated group.

## Discussion

This study examined the 12-month longitudinal changes in physical function among a cohort primarily composed of community-dwelling oldest-old individuals (median age 85.0 [80.5-89.0] years) who participated in 105-minute sessions of a function- focused day-service training program at least once weekly. Participants were stratified into three groups: Improved, Maintained, and Deteriorated, based on the 12-month change in their total SPPB scores.

The most notable finding was that 36.6% of participants improved and 34.4% maintained their SPPB scores, indicating that 71% achieved favorable outcomes over 12 months; furthermore, the overall median SPPB total score remained stable, with no significant improvement or deterioration. These results suggest that physical function- focused training may be associated with maintaining lower-limb function among very old, community- dwelling elderly individuals.

Previous longitudinal studies have reported that SPPB scores decline steadily with aging, particularly during the period preceding death, termed “terminal decline” (TD) phenomenon. Stolz et al.^25)^ reported that in adults aged 70 years and older, SPPB score decreased by approximately 0.4 points per year before the onset of TD, with a sharp decline of 2.9 points during the final year of life (mean age at death 86.8 ± 6.3 years). Similarly, Handing et al.^26)^ reported in their cohort study that community-dwelling elderly individuals (mean age, 73.7 ± 2.9 years) experienced a decline in SPPB scores from a mean of 10.2, decreasing by approximately 0.55 points per year. Compared with these findings, the stable SPPB scores observed in our cohort (median age 85.0 years; median SPPB score 10.0) are remarkable, suggesting that weekly function-focused training may attenuate age-related functional decline of very old community-dwelling elderly and contribute to the maintenance of mobility and independence.

When stratified by SPPB-change groups, the Maintained group showed the highest median baseline SPPB score (12 points), which may reflect a ceiling effect, making further improvement less detectable^2)^. Given that these participants already had high functional capacity, maintaining function itself can be considered a meaningful positive outcome of training.

On the other hand, approximately 60% of participants were excluded or dropped out, resulting in a reduction from 245 to 93 individuals. This raises the possibility of a healthy participant bias, whereby those who were able to participate in the function- focused day service for 12 months may represent a subset with better baseline health and higher motivation. Consequently, the observed findings may overestimate the apparent benefits and should be interpreted with caution.

Interestingly, despite similar baseline SPPB total scores (9.5 vs. 10.0) between the Improved and Deteriorated groups, their 12-month trajectories diverged substantially. This observation may reflect unmeasured factors beyond SPPB. One possible hypothesis is that differences in physiological reserve contributed to these divergent outcomes^27-29)^; however, this construct was not directly assessed in the present study. Supporting this interpretation, grip strength tended to be higher in the Maintained and Improved groups and declined significantly only in the Deteriorated group. Given that grip strength is often considered a marker of overall physical reserve^30, 31)^, these findings may be consistent with, but do not confirm, the role of physiological reserve.

In the analysis of SPPB sub-scores, balance and chair stand performance improved significantly in the Improved group, whereas gait speed did not. A primary explanation for this lack of change in gait speed is a prominent ceiling effect; notably, the median baseline gait sub-score was already 4.0 (the maximum score) across all groups (Improved, Maintained, Deteriorated group), leaving virtually no room for measurable improvement within the SPPB scale. This phenomenon is further compounded by the unequal distribution of task-specific practice, which is reflected in the observed pattern. Previous studies have shown that targeted balance, gait function, and sit-to-stand function interventions effectively improve these specific functions in community-dwelling elderly^32-34)^. In this study, the training regimen was principally oriented towards enhancing balance and strength in the lower limbs with the objective of promoting safety. Conversely, the walking-related practice was restricted. In addition, the 4-m gait speed test is a short-distance assessment in which testing procedures (start type, distance) can meaningfully influence measured speed. Furthermore, age-related "brakes" such as fear of falling further complicate gains^35, 36)^. In fact, our data showed that, among participants with high adherence, the majority successfully preserved their gait speed despite the inherent risks of functional decline. Although the absence of measurable improvement may reflect a ceiling effect and limited practice, the maintenance of performance may still be clinically meaningful. Gait speed is a critical prognostic marker^37)^, and the preservation of it while improving other domains should be interpreted as a favorable response^35-38)^.

Finally, the training program implemented in this study (comprising approximately 105 minutes per session, performed at least once per week) has important practical implications. Although national and international guidelines, such as those from the Japan Ministry of Health and the World Health Organization, recommend a minimum of 150 minutes of moderate-intensity aerobic activity per week along with at least two sessions of strength and balance training^39, 40)^, our findings suggest that even lower-intensity and lower-frequency training may be associated with the maintenance of physical function over 12 months among community-dwelling oldest-old individuals.

## Limitations

This study has several limitations. First, as a single-center retrospective study conducted in a specific day-service setting in Japan, the generalizability of the findings is limited and may not extend to other healthcare systems, independent elderly populations, or higher-frequency exercise programs. Second, the study lacked a non-training control group, making it difficult to clearly distinguish associations related to the training intervention itself from those related to aging or other external factors. Third, there were minor annual variations in training content and staff composition, which could not be completely standardized. Fourth, detailed information regarding participants' total daily activity levels—including physical activities outside day services and voluntary home training habits—as well as their nutritional status, medical history, and comorbidities, was not collected. Consequently, it was impossible to analyze the results while accounting for these factors. Fifth, although all participants understood the assessment procedures, cognitive function was not systematically or longitudinally assessed. Additionally, the significant decrease in sample size from 245 candidates to 93 participants analyzed introduces the possibility of selection bias. A total of 152 individuals were excluded or dropped out of the study, including 9 aged 64 or younger, 83 with missing 12-month follow-up measurements, and 60 unable to continue the 12- month program. The absence of documented reasons for discontinuation suggests that the final cohort may represent a subset of older adults who are more motivated or have superior baseline health status. Considering these limitations, future studies should include large-scale, multicenter prospective designs and incorporate comprehensive assessments of external and behavioral factors.

## Conclusion

In conclusion, community-dwelling elderly individuals (median age, 85.0 [80.5-89.0] years) who continuously participated in a physical function- focused training program at least once per week for 12 months maintained their lower-limb function, as indicated by a stable median SPPB score (from 10.0 [7.0-12.0] to 10.0 [7.5-12.0]). Among participants, 36.6% improved and 34.4% maintained their SPPB scores. These findings suggest that low- frequency, low-intensity function-focused training performed at least once per week may help attenuate age-related functional decline and contribute to maintaining mobility and independent living, even among community-dwelling oldest-old individuals. Future multicenter prospective studies are needed to determine optimal training frequency and program structure, as well as to refine future care-prevention policies for the elderly.

## Data availability

The datasets generated and/or analyzed during the current study are not publicly available due to confidentiality agreements with participants and ethical restrictions imposed by the Ethics Review Committee of Juntendo University. However, de- identified data are available from the corresponding author upon reasonable request, subject to ethics committee approval.

## Author contributions

MK and TF led the study, designed the study, performed data collection and analysis, and wrote the manuscript. AI, MM, MT, RI, FW, AT, and KH provided advice about data analysis and conducted a thorough review and revision of the manuscript. All authors read and approved the final manuscript.

## Conflicts of interest statement

TF reports that Juntendo University has an academic advisory agreement with Takashimaya Co., Ltd. (operator of Takashimaya Your Terrace Futako-Tamagawa); institutional payments were made to Juntendo University, and no personal fees were received by TF. Takashimaya Co., Ltd. had no role in the study design, data analysis, interpretation of results, manuscript preparation, or the decision to submit this article. All other authors declare no conflicts of interest.
